# Beyond AI Literacy: Understanding Health Care Workforce AI Enablement in the Generative AI Era

**DOI:** 10.2196/107290

**Published:** 2026-10-05

**Authors:** Chung-Feng Liu, Yen-Ling Ko

**Affiliations:** 1 Department of Medical Research, Chi Mei Medical Center, Tainan, Taiwan; 2 Department of Internal Medicine, Chi Mei Medical Center, 901 Zhonghua Rd, Yongkang District, Tainan, 710402, Taiwan, 886 62812811 ext 52590; 3 General Education Center, Chia Nan University of Pharmacy and Science, Tainan, Taiwan

**Keywords:** artificial intelligence, AI, artificial intelligence literacy, AI literacy, artificial intelligence fluency, AI fluency, artificial intelligence harnessing, AI harnessing, health care workforce, artificial intelligence enablement, AI enablement

## Abstract

The rapid diffusion of generative AI is transforming health care delivery, education, administration, and research. In response, health care organizations have invested heavily in AI literacy initiatives and workforce development programs. Existing frameworks primarily focus on whether health care professionals understand AI and whether they can engage with AI effectively. However, health care practice increasingly reveals that individuals with similar levels of AI literacy and AI engagement often contribute very differently to AI-enabled work and organizational adoption. Some professionals primarily use AI to improve their own work, whereas others facilitate AI adoption, coordinate stakeholders, and integrate AI into routine practice. This observation suggests that current perspectives may overlook an important dimension of workforce AI enablement. In this Viewpoint, we argue that AI literacy and AI engagement alone provide an incomplete explanation of how health care organizations realize the benefits of AI. Drawing upon literature from AI literacy, fluency theory, human-AI interaction, innovation diffusion, implementation science, and health care workforce development, we propose the health care workforce AI enablement matrix (HWAEM). HWAEM conceptualizes workforce AI enablement through 2 complementary capabilities: AI fluency and AI harnessing. AI fluency refers to the capability to engage with AI effectively, appropriately, and responsibly across professional contexts, whereas AI harnessing refers to the capability to identify opportunities for AI-enabled improvement, mobilize stakeholders, facilitate adoption, and integrate AI into collective work practices. The interaction of these capabilities generates 4 workforce profiles: AI novices, AI practitioners, AI facilitators, and AI leaders. Through HWAEM, this Viewpoint argues that health care workforce AI enablement is better understood through the complementary capabilities of AI fluency and AI harnessing than through AI literacy and AI engagement alone. We illustrate how these profiles manifest in health care practice and discuss implications for workforce development and AI implementation. HWAEM offers a new perspective for understanding health care workforce preparedness in the generative AI era and provides a foundation for future empirical research.

## Introduction

Generative AI is rapidly becoming embedded in health care work. Large language models (LLMs) are increasingly used to support clinical documentation, patient education, information retrieval, administrative tasks, research activities, and clinical decision support. Unlike previous generations of health care AI that were often confined to specialized applications, generative AI is now directly accessible to frontline health care professionals and is increasingly integrated into routine health care practice. As a result, AI adoption can occur at the individual level long before formal organizational implementation. This creates a growing gap between effective personal AI use and successful integration into collective practice.

In response, health care organizations have increasingly recognized the importance of AI literacy, competency frameworks, and workforce training initiatives. Existing discussions of workforce preparedness are largely dominated by concepts such as AI literacy, AI competency, AI readiness, and AI skills [1,2]. Collectively, these perspectives focus on whether health care professionals understand AI and whether they can use AI appropriately, effectively, and responsibly.

These efforts remain important. Recent evidence suggests that future health care professionals require not only AI-related knowledge but also capabilities in critical evaluation, ethical reasoning, communication, and interdisciplinary collaboration [3]. Similarly, emerging health care literature has highlighted persistent gaps between AI literacy, workforce preparedness, and meaningful AI integration into clinical practice, suggesting that education alone may be insufficient for successful AI adoption [4]. At the same time, international organizations have highlighted that AI is reshaping health care workforce roles, skill requirements, and models of care delivery. The Organisation for Economic Co-operation and Development (OECD) has highlighted that AI is transforming professional roles and skill requirements across health occupations, creating a growing need for workforce adaptation, training, and new forms of AI-enabled work [5]. More recently, an OECD analysis of approximately 55.5 million online job postings revealed substantial variation in digital and AI skill demand across health occupations, underscoring the need for differentiated workforce development strategies rather than a one-size-fits-all approach [6]. Similarly, the World Health Organization (WHO) has identified workforce readiness, stakeholder engagement, and organizational integration as critical determinants of successful AI adoption within health systems [7].

However, health care organizations increasingly observe that professionals with similar levels of AI literacy and AI use often contribute very differently to AI-enabled work. Some health care professionals use AI frequently and skillfully yet generate limited impact beyond personal productivity gains. Others, despite possessing only moderate technical AI expertise, successfully identify opportunities for AI adoption, coordinate stakeholders, facilitate implementation, and help integrate AI into routine practice.

This phenomenon raises an important question: if health care professionals possess comparable levels of AI literacy and AI use, why do their contributions to AI-enabled practice and organizational adoption differ so substantially? Existing frameworks explain whether health care professionals understand AI and whether they use AI, but they provide limited insight into why some individuals become catalysts for AI-enabled change while others do not.

To address this gap, we propose the health care workforce AI enablement matrix (HWAEM), a conceptual framework integrating 2 complementary workforce capabilities: AI fluency and AI harnessing. The conceptualization of AI fluency was informed by research on AI literacy [1,2], the emerging AI fluency framework [8], and human-AI interaction [9]. AI harnessing was informed by innovation diffusion [10] and implementation research in health service organizations [11]. Health care workforce studies and policy reports provided the health care context by highlighting changing skill requirements, workforce readiness, and implementation capacity [5-7]. To facilitate comparison, Table 1 summarizes the conceptual distinctions among AI literacy, AI fluency, and AI harnessing, providing the conceptual foundation for the proposed HWAEM. This Viewpoint aims to (1) explain why AI literacy and AI engagement alone are insufficient to explain health care workforce AI enablement, (2) introduce AI fluency and AI harnessing as complementary capabilities, (3) propose 4 workforce profiles, and (4) discuss their implications for workforce development and AI implementation. This Viewpoint is intended for health care leaders, clinicians, educators, implementation researchers, health informaticians, and policymakers involved in health care workforce development and AI implementation.

**Table 1.** Conceptual comparison of AI literacy, AI fluency, and AI harnessing.

Dimensions	AI literacy	AI fluency	AI harnessing
Definition	Understanding AI concepts and capabilities	Engaging with AI effectively, appropriately, and responsibly	Facilitating AI adoption and integration into collective practice
Primary focus	Individual knowledge	Individual capability	Team or organization
Key question	Do I understand AI?	Can I work effectively with AI?	How can we enable AI in collective practice?
Primary contribution	Foundational AI understanding	Effective human-AI collaboration	Organizational AI enablement

## Why AI Literacy and AI Use Are Not Enough

AI literacy has emerged as one of the most influential concepts in contemporary AI education. Long and Magerko [1] defined AI literacy as a set of competencies that enable individuals to critically evaluate, communicate with, and interact with AI systems. Subsequent work by Ng et al [2] further conceptualized AI literacy as encompassing knowledge, application, evaluation, ethics, and awareness related to AI technologies.

Within health care, AI literacy has become a central focus of workforce preparation. Recent reviews emphasize the need for health care professionals to understand AI capabilities and limitations, critically evaluate AI outputs, recognize ethical implications, and communicate effectively about AI-enabled technologies [3]. These competencies remain essential because health care professionals ultimately retain responsibility for decisions affecting patient care.

Despite their differences, most AI literacy frameworks share a common focus: helping individuals understand AI and interact with it appropriately [1,2]. In essence, AI literacy addresses a fundamental question: Do I understand AI?

However, as generative AI becomes increasingly embedded in routine health care work, organizations face a second question: Can I work effectively with AI?

Understanding AI and working effectively with AI are related but distinct capabilities. An individual may understand AI concepts yet struggle to integrate AI into daily work. Conversely, another individual may demonstrate highly effective AI-assisted performance despite possessing only modest theoretical knowledge.

Similarly, AI use alone may not adequately explain workforce AI enablement. Measures of AI use typically focus on the frequency or extent of use but provide limited insight into how AI is used, whether outputs are critically evaluated, or whether AI contributes to broader organizational adoption. Similar concerns have been raised in health care AI research, where positive attitudes toward AI and increasing levels of AI use do not necessarily translate into routine clinical integration or organizational adoption [12].

More fundamentally, AI use cannot distinguish between using AI to improve one’s own work and enabling AI adoption across a team or organization. For example, a physician may use generative AI extensively to improve personal productivity, whereas a nurse manager may use AI less frequently but successfully identify opportunities for AI adoption, coordinate clinicians and information technology staff, and facilitate AI integration into routine workflows. Despite using AI less frequently, the latter may contribute far more substantially to organizational AI adoption. This distinction suggests that AI use alone cannot fully explain health care workforce AI enablement and provides the conceptual rationale for distinguishing AI fluency from AI harnessing.

Recent workforce evidence further suggests that AI-related skill requirements are heterogeneous rather than uniform. An OECD analysis of approximately 55.5 million online job postings identified substantial variation in digital and AI skill demand across health occupations, highlighting the need for differentiated workforce development rather than a single model of AI competence [6].

Consequently, AI literacy and AI use remain necessary foundations for workforce preparedness. However, they may be insufficient for explaining why health care professionals differ substantially in their ability to leverage AI in practice and contribute to AI-enabled change.

## AI Fluency: Effective Engagement With AI

Although AI literacy provides an essential foundation for workforce preparedness, understanding AI does not necessarily translate into effective engagement with AI. As generative AI becomes increasingly embedded in professional work, emerging discussions have begun to distinguish AI literacy from AI fluency. Recent initiatives such as the AI fluency framework emphasize that AI fluency involves engaging with AI systems in ways that are effective, efficient, ethical, and safe, extending beyond knowledge of AI technologies alone [8].

This perspective is particularly relevant in health care, where professionals must not only understand AI capabilities and limitations but also engage with AI responsibly in contexts involving patient care, clinical decision-making, education, research, and health care operations.

This emerging view is consistent with broader theories of fluency. Binder [13] described fluency as the integration of accuracy and efficiency that enables successful performance in authentic environments, while Segalowitz [14] emphasized the ability to mobilize knowledge rapidly, appropriately, and consistently during task execution. Together, these perspectives suggest that fluency extends beyond knowledge acquisition and reflects the capability to apply and adapt knowledge effectively in practice.

Research on human-AI interaction further supports this distinction. Amershi et al [9] proposed guidelines emphasizing users’ abilities to understand system capabilities, monitor outputs, identify errors, adjust interactions, and maintain appropriate oversight. These capabilities extend beyond AI knowledge and reflect the practical quality of human-AI engagement.

Importantly, AI literacy and AI fluency address different questions. AI literacy primarily concerns whether individuals understand AI, whereas AI fluency concerns whether they can engage with AI effectively, appropriately, and responsibly when AI is available.

Recent evidence further suggests that health occupations require different combinations of digital, analytical, and AI-related skills rather than a uniform level of technical expertise [6]. Effective AI-enabled work therefore depends not only on knowledge acquisition but also on the ability to engage with AI adaptively across diverse professional tasks, responsibilities, and contexts.

Building upon these foundations, we introduce AI fluency as a workforce capability distinct from AI literacy. AI fluency refers to the capability to engage with AI effectively, appropriately, and responsibly across professional contexts. It reflects more than familiarity with AI tools or the frequency of use. Rather, it encompasses recognizing opportunities where AI may be useful, communicating effectively with AI systems, critically evaluating outputs, refining interactions, adapting AI assistance to specific tasks, and maintaining professional judgment, accountability, and oversight.

In health care settings, AI fluency may include engaging with generative AI to support clinical documentation, patient education, literature review, knowledge retrieval, educational activities, research, administrative work, or decision support. Importantly, high AI fluency does not necessarily imply broader organizational adoption. A health care professional may demonstrate sophisticated AI engagement while remaining cautious about extending AI into routine clinical workflows because of concerns regarding reliability, privacy, patient safety, accountability, regulatory requirements, or workflow disruption.

AI fluency therefore represents a shift from understanding AI toward effective engagement with AI. It emphasizes what health care professionals can do with AI rather than simply what they know about AI.

Nevertheless, health care organizations often observe that professionals with similar levels of AI fluency contribute differently to AI-enabled practice and organizational adoption. This observation suggests that effective engagement with AI alone may not fully explain workforce AI enablement.

Examples of observable behaviors associated with AI fluency may include recognizing opportunities where AI may be useful, refining prompts, critically evaluating AI-generated outputs, adapting AI assistance to different professional tasks, and maintaining appropriate human judgment, accountability, and oversight.

## AI Harnessing: Facilitating AI Integration Into Collective Practice

Health care organizations frequently encounter an intriguing phenomenon. Some professionals engage with AI extensively and skillfully yet exert little influence on broader AI adoption. Others become catalysts for AI-enabled change despite only moderate levels of AI engagement. This phenomenon cannot be fully explained by AI literacy or AI fluency alone.

Consider 2 health care professionals with comparable levels of AI literacy. Both recognize the potential of AI and are willing to engage with AI in their professional work. Yet their contributions to AI-enabled change may differ substantially. One physician may use AI extensively to improve personal productivity through literature review, clinical documentation, research, or educational activities. Another professional may engage with AI less frequently but actively identify opportunities for AI-enabled improvement, coordinate stakeholders, facilitate implementation efforts, and help integrate AI into routine workflows.

From the perspective of AI fluency alone, these individuals may appear similar in their ability to engage effectively with AI. From an organizational perspective, however, their contributions are fundamentally different. One primarily improves individual performance, whereas the other helps embed AI into collective practice. This observation suggests that effective engagement with AI alone may be insufficient for explaining workforce AI enablement.

Research on innovation diffusion and implementation has consistently demonstrated that successful innovation depends not only on technological capability but also on the ability to facilitate adoption within a social system. Rogers [10] emphasized the importance of communication channels, opinion leaders, and change agents in the diffusion of innovations. Similarly, Greenhalgh et al [11] highlighted the critical roles of stakeholder engagement, contextual adaptation, implementation processes, and organizational support in determining whether innovations become embedded in routine practice. Recent research in professional services further suggests that realizing AI value depends on how organizations harness AI affordances while addressing contextual and organizational constraints [15].

These issues have become increasingly visible in contemporary health care AI policy. WHO Europe identifies workforce readiness, stakeholder engagement, governance, and implementation capacity as integral components of health system–AI integration [7]. More recently, the OECD has framed the scaling of AI in health as an implementation and system capacity challenge rather than simply a matter of technological availability [16]. Together, these perspectives suggest that realizing the benefits of AI depends not only on technological performance but also on the ability to integrate AI into work processes and organizational activities.

Collectively, this body of literature highlights that enabling AI in health care requires capabilities beyond individual AI use, including stakeholder engagement, workflow redesign, governance, implementation processes, and organizational learning. Drawing on these insights, we introduce AI harnessing as a distinct workforce capability. AI harnessing refers to the capability to identify opportunities for AI-enabled improvement, mobilize stakeholders, facilitate adoption, and integrate AI into collective work practices.

Although AI fluency focuses on effective engagement with AI at the individual level, AI harnessing focuses on facilitating the incorporation of AI into shared workflows, professional practices, and organizational activities. Importantly, AI harnessing is not synonymous with implementation success, digital transformation, organizational performance, or value creation. Rather, it represents a workforce capability that supports the adoption and integration of AI within collective practice.

Health care professionals with strong AI harnessing capabilities may identify workflow challenges suitable for AI support, coordinate implementation efforts, engage stakeholders, address concerns from frontline users, facilitate learning, and help normalize AI-assisted work practices within teams. Notably, they are not necessarily the most proficient AI users. Their contribution lies in helping others adopt and integrate AI successfully within everyday health care practice.

AI harnessing therefore represents a shift from individual AI engagement toward collective AI integration. However, health care professionals vary considerably in the extent to which they demonstrate AI fluency and AI harnessing. Understanding these variations may provide a richer perspective on workforce AI enablement.

Examples of observable behaviors associated with AI harnessing may include identifying opportunities for AI-enabled improvement, engaging multidisciplinary stakeholders, coordinating implementation activities, redesigning workflows, facilitating AI adoption, aligning AI initiatives with organizational governance, and promoting organizational learning.

## HWAEM

### Overview

We propose HWAEM as a conceptual framework for understanding how health care professionals contribute to AI-enabled clinical practice and organizational implementation.

HWAEM integrates two complementary workforce capabilities:

1. AI fluency—the capability to engage with AI effectively, appropriately, and responsibly across diverse health care professional contexts
2. AI harnessing—the capability to facilitate AI integration into multidisciplinary clinical workflows and collective health care practice

Unlike competency ladders, capability maturity models, or developmental stage frameworks, HWAEM is not intended to represent a linear progression from lower to higher levels of expertise. Instead, it functions as a conceptual workforce typology that describes different patterns of workforce AI enablement in health care.

Together, AI fluency and AI harnessing provide a 2D perspective on workforce AI enablement. Different combinations of these capabilities give rise to 4 proposed workforce profiles: AI novices, AI practitioners, AI facilitators, and AI leaders. As illustrated in Figure 1, each profile reflects a distinct pattern of AI enablement rather than a fixed personality type, developmental stage, or empirically validated category.

**Figure 1.**
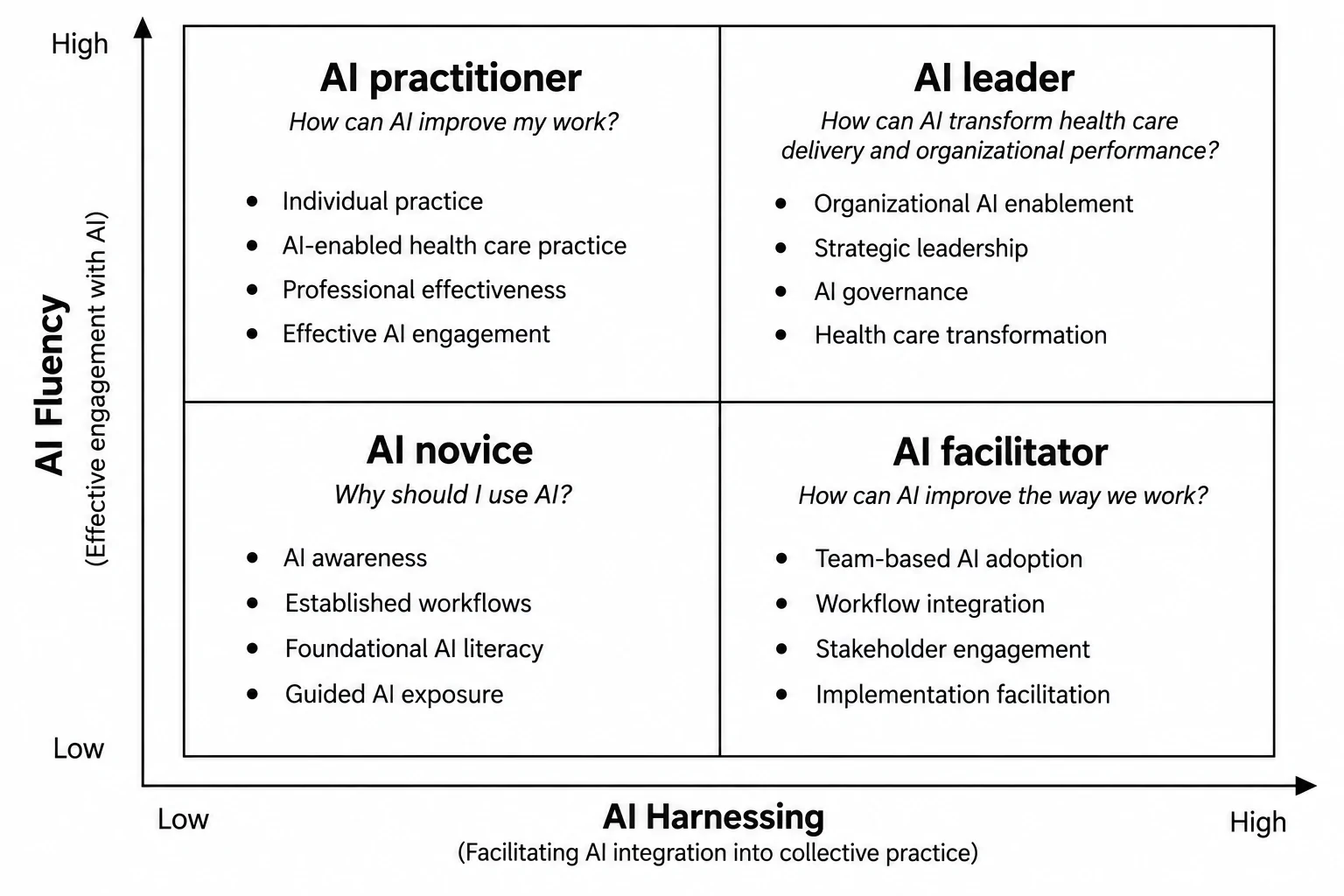
Health care workforce AI enablement matrix (HWAEM). HWAEM is a conceptual workforce typology rather than a competency ladder, capability maturity model, or developmental stage framework. The 4 proposed workforce profiles illustrate distinct patterns of health care workforce AI enablement and should not be interpreted as fixed personality types, developmental stages, or empirically validated categories.

### AI Novices

AI novices represent a proposed workforce profile characterized by relatively limited AI engagement in both individual and collective practice. AI has not yet become a meaningful part of their daily professional activities, and health care work continues to rely primarily on established clinical knowledge, professional experience, and conventional workflows.

Their limited engagement may reflect unfamiliarity with AI, uncertainty regarding its value, concerns about reliability or accountability, or simply limited opportunities for exposure. Consequently, AI remains largely peripheral to their approach to patient care and health care work.

Importantly, this profile does not imply resistance to AI. Rather, it reflects the current absence of a clear rationale for incorporating AI into professional practice.

Accordingly, AI novices primarily ask, Why should I use AI?

### AI Practitioners

AI practitioners actively engage with AI and view it as a tool for enhancing their own professional effectiveness. They frequently explore how AI can support clinical documentation, literature review, patient education, research, learning, administrative work, and other professional tasks.

Their primary interest is not organizational adoption but personal practice improvement. They focus on how AI can help them work more efficiently, make better use of information, reduce routine workload, or enhance the quality of their professional activities. Although they may possess considerable experience interacting with AI, their efforts remain largely centered on individual performance rather than collective implementation.

The defining characteristic of this profile is its emphasis on leveraging AI to improve individual professional practice rather than facilitating broader organizational adoption.

Accordingly, AI practitioners primarily ask, How can AI improve my work?

### AI Facilitators

AI facilitators view AI primarily as a means of improving how health care teams and organizations work. Rather than focusing on personal productivity alone, they are concerned with how AI can support shared workflows, collaboration, communication, and service delivery.

They recognize opportunities for AI-enabled improvement, engage stakeholders, coordinate implementation efforts, address concerns from frontline users, and help integrate AI into routine practice. Importantly, they are not necessarily the most technically proficient AI users. Their contribution lies in enabling others to adopt and use AI successfully within everyday health care work.

The defining characteristic of this profile is its emphasis on enabling AI to move from individual use to collective health care practice through stakeholder engagement, workflow integration, and implementation facilitation.

Accordingly, AI facilitators primarily ask, How can AI improve the way we work?

### AI Leaders

AI leaders view AI through a broader organizational and system-level lens. They are concerned not only with how AI affects individual professionals or teams but also with how it can contribute to health care quality, patient outcomes, workforce capability, organizational performance, and the long-term transformation of health care delivery.

They connect technological opportunities with strategic priorities, governance considerations, workforce development, and implementation efforts. By bridging individual engagement and organizational integration, they help translate AI potential into sustainable health care impact.

The defining characteristic of this profile is its emphasis on aligning AI opportunities with organizational strategy, governance, workforce development, and sustainable health care transformation.

Accordingly, AI leaders primarily ask, How can AI transform health care delivery and organizational performance?

The characteristics of the 4 proposed workforce profiles are summarized in Table 2.

**Table 2.** Characteristics of health care workforce AI enablement matrix workforce profiles.

Workforce profiles	Description	Primary contribution	Development priority
AI novice	AI has not yet become a meaningful part of professional practice. Clinical work continues to rely primarily on established knowledge, experience, and conventional workflows.	Awareness of AI opportunities and limitations, and potential applications	Foundational AI literacy, responsible AI awareness, and guided AI exposure
AI practitioner	Actively engages with AI to enhance personal professional effectiveness in tasks such as documentation, information retrieval, education, research, or administrative work	Individual AI-enabled health care practice improvement	Developing more effective, adaptive, and responsible AI engagement within professional practice
AI facilitator	Focuses on helping teams and organizations adopt and integrate AI into shared workflows and routine practice	Team-based AI adoption, workflow integration, and implementation facilitation	Stakeholder engagement, implementation leadership, workflow redesign, and change facilitation
AI leader	Connects AI opportunities with organizational strategy, workforce development, governance, and health care transformation	Organizational and system-level AI enablement and sustainable impact on health care	Strategic leadership, AI governance, workforce capability development, and organizational transformation

## Real-World Manifestations of HWAEM

### Overview

Although HWAEM is proposed as a conceptual framework, the 4 workforce profiles can be readily observed in contemporary health care settings. These profiles should not be interpreted as fixed categories, personality traits, or developmental stages. Rather, they represent distinct patterns of workforce AI enablement that emerge from different combinations of AI fluency and AI harnessing (Figure 1). Individuals may exhibit different profiles depending on their professional roles, organizational responsibilities, and implementation contexts.

The following scenarios illustrate how HWAEM may manifest in real-world health care practice.

### AI Novice: AI Is Not Yet Part of My Work

A staff nurse has heard about ChatGPT (OpenAI) and attended an introductory AI workshop organized by the hospital. However, AI remains largely outside her daily workflow. Concerns regarding reliability, privacy, professional accountability, and uncertainty about its practical value limit her willingness to experiment with AI-assisted work. Although she recognizes that AI may become increasingly important in health care, she has not yet identified meaningful opportunities to incorporate AI into either her personal work routines or team activities.

AI remains peripheral to both her individual practice and the collective work environment. Her primary challenge is not learning how to use AI but understanding why AI should become part of her professional practice.

### AI Practitioner: AI Improves My Work

A physician routinely uses generative AI to summarize journal articles, prepare presentations, draft patient education materials, and support manuscript development. Through repeated engagement, he has developed effective prompting strategies, critically evaluates AI-generated outputs, and consistently achieves meaningful productivity gains.

However, these practices remain largely personal. He rarely shares workflows with colleagues, participates in implementation initiatives, or actively promotes broader adoption. AI primarily serves as a tool for enhancing his own professional effectiveness rather than improving collective work processes.

For this physician, the value of AI is realized through improving the quality, efficiency, and effectiveness of his own work.

### AI Facilitator: AI Improves the Way We Work

A nurse manager recognizes that documentation requirements consume substantial nursing time and contribute to staff frustration. Although advanced AI use is not the primary focus of her role, she actively explores opportunities for AI-assisted documentation. She coordinates discussions among frontline nurses, information technology staff, and hospital administrators, organizes pilot testing, gathers feedback, and helps refine implementation strategies.

As a result, AI becomes embedded within routine workflows across the unit. Her contribution lies less in personal AI use and more in helping others adopt and integrate AI successfully into everyday practice.

For this manager, the value of AI is realized through improving how people work together and how care processes are delivered.

### AI Leader: AI Transforms Health Care Delivery and Organizational Performance

A chief medical information officer oversees multiple AI initiatives across a health care organization. In addition to engaging effectively with AI in her own work, she develops governance structures, supports workforce development programs, aligns AI initiatives with organizational strategy, and facilitates collaboration among clinicians, administrators, information technology teams, and executive leadership.

Rather than focusing on isolated AI applications, she views AI as part of a broader effort to improve health care delivery, organizational learning, workforce capability, and service innovation. Her role extends beyond implementation to shaping how AI contributes to organizational priorities and long-term transformation.

For this leader, the value of AI is realized through sustainable organizational change and health care transformation at scale.

### Why These Scenarios Matter

These scenarios illustrate why health care workforce AI enablement cannot be fully understood through AI literacy or AI use alone. Health care professionals with similar levels of AI literacy may demonstrate markedly different patterns of AI-enabled behavior and organizational contribution. Likewise, frequent AI use does not necessarily translate into broader adoption, implementation influence, or organizational impact.

Most importantly, HWAEM highlights a distinction that is largely absent from existing AI literacy, competency, and readiness frameworks: the difference between professionals who engage effectively with AI and professionals who facilitate AI integration within collective practice. AI practitioners primarily leverage AI to improve individual performance, whereas AI facilitators help embed AI within collective work processes. Despite possessing comparable levels of AI literacy—and in some cases similar levels of AI fluency—these groups contribute to workforce AI enablement in fundamentally different ways.

More broadly, the 4 profiles illustrate different ways in which health care professionals create value from AI. AI novices are still exploring the relevance of AI to their work. AI practitioners focus on improving individual practice. AI facilitators focus on improving collective practice. AI leaders focus on transforming health care delivery and organizational performance. These differences reflect distinct patterns of workforce AI enablement rather than stages of professional development.

By distinguishing AI fluency from AI harnessing, HWAEM provides a more nuanced perspective on workforce preparedness and offers a potential explanation for why some health care organizations successfully integrate AI into routine practice while others struggle despite widespread access to AI technologies. As health care increasingly shifts from AI experimentation to implementation and scale-up, understanding these different patterns of workforce AI enablement may become as important as understanding AI technologies themselves.

## Implications for Workforce Development and AI Implementation

The rapid adoption of generative AI has prompted substantial investment in AI literacy programs, competency frameworks, and workforce training initiatives. Although these efforts remain important, HWAEM suggests that health care organizations may need to broaden how workforce AI preparedness is conceptualized, assessed, and developed. Figure 1 maps the 4 workforce profiles generated by different combinations of AI fluency and AI harnessing, while Table 2 summarizes their distinct characteristics and developmental priorities.

Existing workforce development strategies often assume that increasing AI literacy and encouraging AI use will naturally lead to greater organizational adoption and impact. However, HWAEM suggests that workforce AI enablement involves more than knowledge acquisition or individual tool use. Health care professionals with similar levels of AI literacy may contribute very differently to AI-enabled practice, and frequent AI use does not necessarily translate into implementation influence or organizational change.

This perspective aligns with implementation research demonstrating that successful innovation depends not only on individual competence but also on stakeholder engagement, workflow integration, contextual adaptation, and organizational support [11]. Recent health care AI policy reports similarly identify workforce readiness, implementation capacity, governance, and organizational integration as critical determinants of successful AI adoption [7,16].

Importantly, HWAEM suggests that workforce development should not be viewed as a one-size-fits-all process. Different workforce profiles may require different developmental priorities, and AI fluency and AI harnessing may also require different approaches to assessment, reflecting not only effective AI use but also contributions to implementation and organizational integration. AI novices may benefit from foundational AI literacy and guided exposure. AI practitioners may require support in developing more effective and responsible AI engagement. AI facilitators may benefit from implementation, stakeholder engagement, and change facilitation capabilities. AI leaders may require competencies related to governance, workforce strategy, and organizational transformation.

Accordingly, health care organizations may need to develop not only AI users but also AI enablers. Rather than relying solely on the frequency or sophistication of AI use, organizations may identify potential AI facilitators based on their demonstrated ability to identify opportunities for AI-enabled improvement, engage multidisciplinary stakeholders, coordinate implementation initiatives, and facilitate AI integration into routine practice. One particularly important implication of HWAEM is the recognition of AI facilitators as a distinct and potentially underappreciated workforce group. These individuals help connect technological opportunities with people, workflows, and organizational priorities, enabling AI to move from isolated experimentation to routine practice. Their role resembles that of change agents and implementation facilitators described in the innovation literature [10,11].

From this perspective, workforce development should extend beyond AI literacy and technical proficiency to include capabilities associated with AI harnessing, such as opportunity recognition, stakeholder engagement, implementation facilitation, workflow redesign, organizational learning, and implementation governance.

More broadly, HWAEM highlights that successful AI implementation depends not only on trustworthy technologies, technical performance, or governance structures but also on workforce capabilities that support effective, safe, and sustainable integration into routine practice. Recent WHO and OECD reports similarly emphasize that trustworthy AI adoption depends not only on governance mechanisms but also on workforce readiness and implementation capacity [7,16]. HWAEM therefore suggests that workforce development and AI implementation should be viewed as complementary priorities rather than separate organizational initiatives.

## Limitations and Future Research

HWAEM is proposed as a conceptual workforce typology and therefore requires empirical validation. Future research should examine whether AI fluency and AI harnessing can be operationalized and measured as distinct workforce capabilities and whether the 4 workforce profiles proposed by HWAEM emerge empirically in real-world health care populations.

Importantly, workforce AI enablement may manifest differently across health care professions and practice settings. Physicians, nurses, allied health professionals, administrators, educators, and health informaticians may require different combinations of AI fluency and AI harnessing depending on their clinical responsibilities, workflow characteristics, and organizational roles. Future studies should therefore examine how workforce AI enablement profiles vary across professional groups, health care settings, organizational contexts, and stages of AI implementation.

Future research should also investigate whether AI fluency and AI harnessing contribute differently to outcomes that are particularly relevant to health care organizations, including AI adoption, implementation success, workforce sustainability, technostress, professional well-being, quality improvement, organizational learning, and responsible AI implementation.

More importantly, future studies should evaluate whether HWAEM provides explanatory value beyond existing concepts such as AI literacy, competency, readiness, technology acceptance, and digital capability. Demonstrating such incremental explanatory value will be essential for determining whether distinguishing AI fluency from AI harnessing meaningfully advances understanding of workforce AI enablement in health care.

## Conclusions

Health care organizations are rapidly moving from AI experimentation toward implementation and scale-up. In this context, the challenge is no longer simply whether health care professionals understand AI or use AI, but whether health care systems can successfully integrate AI into routine clinical and operational practice.

HWAEM proposes that workforce AI enablement involves 2 complementary capabilities: AI fluency, reflecting effective engagement with AI, and AI harnessing, reflecting the facilitation of AI integration within collective practice. By distinguishing these capabilities, HWAEM highlights an often-overlooked reality of health care AI adoption: some professionals primarily improve their own work through AI, whereas others help embed AI into the shared work of health care.

More broadly, HWAEM suggests that workforce AI enablement is not a single continuum of AI competence but a set of distinct patterns through which health care professionals create value from AI. Understanding these patterns may help health care organizations better align workforce development, implementation capacity, and responsible AI initiatives.

Ultimately, the future success of health care AI may depend not only on the intelligence of AI systems but also on the ability of health care workforces to engage with and harness AI in ways that improve care, support professionals, and strengthen health systems.

## Abbreviations

HWAEM: health care workforce AI enablement matrix
LLM: large language model
OECD: Organisation for Economic Co-operation and Development
WHO: World Health Organization
